# RBM6-RBM5 transcription-induced chimeras are differentially expressed in tumours

**DOI:** 10.1186/1471-2164-8-348

**Published:** 2007-10-01

**Authors:** Ke Wang, Gino Ubriaco, Leslie C Sutherland

**Affiliations:** 1 Tumour Biology Group, Regional Cancer Program of the Sudbury Regional Hospital, Sudbury, Ontario, Canada; 2 Northern Ontario School of Medicine, Sudbury, Ontario, Canada; 3 Biomolecular Sciences Program, Laurentian University, Sudbury, Ontario, Canada; 4 Department of Respiratory Medicine, The Second Affiliated Hospital of Jilin University, Changchun, Jilin, China

## Abstract

**Background:**

Transcription-induced chimerism, a mechanism involving the transcription and intergenic splicing of two consecutive genes, has recently been estimated to account for ~5% of the human transcriptome. Despite this prevalence, the regulation and function of these fused transcripts remains largely uncharacterised.

**Results:**

We identified three novel transcription-induced chimeras resulting from the intergenic splicing of a single RNA transcript incorporating the two neighbouring 3p21.3 tumour suppressor locus genes, *RBM6 *and *RBM5*, which encode the RNA Binding Motif protein 6 and RNA Binding Motif protein 5, respectively. Each of the three novel chimeric transcripts lacked exons 3, 6, 20 and 21 of RBM6 and exon 1 of RBM5. Differences between the transcripts were associated with the presence or absence of exon 4, exon 5 and a 17 nucleotide (nt) sequence from intron 10 of RBM6. All three chimeric transcripts incorporated the canonical splice sites from both genes (excluding the 17 nt intron 10 insertion). Differential expression was observed in tumour tissue compared to non-tumour tissue, and amongst tumour types. In breast tumour tissue, chimeric expression was associated with elevated levels of RBM6 and RBM5 mRNA, and increased tumour size. No protein expression was detected by *in vitro *transcription/translation.

**Conclusion:**

These results suggest that RBM6 mRNA experiences altered co-transcriptional gene regulation in certain cancers. The results also suggest that RBM6-RBM5 transcription-induced chimerism might be a process that is linked to the tumour-associated increased transcriptional activity of the *RBM6 *gene. It appears that none of the transcription-induced chimeras generates a protein product; however, the novel alternative splicing, which affects putative functional domains within exons 3, 6 and 11 of RBM6, does suggest that the generation of these chimeric transcripts has functional relevance. Finally, the association of chimeric expression with breast tumour size suggests that RBM6-RBM5 chimeric expression may be a potential tumour differentiation marker.

## Background

Transcription-induced chimerism, resulting from the transcription and intergenic splicing of two consecutive genes, was previously thought to be a rare event in mammals. Recent studies, however, incorporating systematic *in silico *analyses of ESTs and cDNAs in the NCBI databases, conclude that as much as 5% of the human transcriptome is comprised of chimeric sequences [1]. These fusion transcripts are generated from tandem genes that are physically located within ~50 kb of each other, the median distance being ~8.5 kb [2]. Although transcription-induced chimeras can function to (1) expand functional protein diversity, (2) alter transcriptional regulation, (3) inhibit transcription of the two participating genes, or (4) inhibit transcription of putative functional intergenic sequences (such as small miRNA sequences), the mechanism regulating its occurrence remains elusive [2].

*RBM6 *(RNA Binding Motif protein 6) [GenBank Accession Number: NM_005777] was first identified by positional cloning from a small cell lung carcinoma homozygous deletion region at the 3p21.3 tumour suppressor locus [3], and, in parallel, as a differentially expressed transcript during granulocyte differentiation [4]. The gene covers ~137 kilobases (kb) and has 21 exons. *RBM6 *is immediately adjacent to, telomeric to, and 11 kb from, the *RBM5 *gene. While the *RBM6 *gene has been shown to be either deleted or disrupted in some lung cancers [5], RBM6 mRNA was recently found to be significantly upregulated in breast cancer [6]. In addition, the RBM6 protein was first isolated in an autologous antibody screen from a patient with adenocarcinoma of the lung, demonstrating an association between elevated levels of RBM6 protein and cancer [7]. Of significance to the work reported herein, a novel trans-fusion protein incorporating the amino-terminal region of RBM6 (breaking 21 amino acids into exon 3) with the carboxy-terminal region of colony stimulating factor 1 receptor (CSF1R) was recently reported in acute megakaryoblastic leukemia [8].

RBM6 pre-mRNA is alternatively spliced to produce at least five variants [3,7,9]. RBM6A, B, C and D differ only in relation to which alternate sequence from intron 2 is incorporated between exons 2 and 3. A fifth splice variant, RBM6Δ6, is identical to the predominant transcript, RBM6A, but lacks exon 6. Timmer and colleagues [3] demonstrated that expression of this RBM6Δ6 transcript was much higher in normal lung tissue than in lung cancer tissues, suggesting that removal of exon 6, which contains one of two consensus RNA recognition motif (RRM) domains within the protein, is important for tumour suppression. It was recently reported that expression of either RBM6 or RBM6Δ6 mRNA and RBM10v2 mRNA (encoding a protein with ~30% identity to both RBM6 and RBM5), was downregulated and highly correlated in relation to a number of clinicopathologic parameters normally associated with poor breast cancer prognosis, suggesting that the coordinated expression, and/or alternative splicing, of RBM6 and RBM10v2 is an important aspect of breast tumorigenesis [10].

The *RBM5 *(RNA Binding Motif protein 5)/*LUCA-15/H37 *gene covers approximately 30 kb of genomic DNA and has 25 exons. *RBM5 *[GenBank Accession Number: NM_005778] generates at least four RNA splice variants, RBM5, RBM5Δ6, RBM5+6 and RBM5+5+6 [9]. All of these transcripts are ubiquitously expressed, albeit to differing levels, in normal tissues. Expression of RBM5 mRNA is downregulated in tumour tissue compared to normal tissue [11-16], although our recent study reports that expression of RBM5 mRNA is marginally upregulated (*p *= 0.063) and protein is significantly upregulated (*p *= 1.43 × 10^-8^) in breast tumour tissue [6]. Numerous functions have been ascribed to *RBM5 *gene products, including tumor suppression [11,17], apoptosis modulation [17-19], cell cycle regulation [20] and RNA binding [5,12], however, the mechanism of action of the full-length RBM5 protein is only just beginning to be delineated [17].

In the study described herein, we set out to investigate the existence of an RBM6-RBM5 chimeric transcript whose expression, or protein product, regulated expression of *RBM6 *and *RBM5*. Here we report the unexpected identification of not one but three novel non-coding RBM6-RBM5 chimeric mRNA transcripts, which were differentially expressed in tumour versus non-tumour tissue and whose expression was not associated with decreased RBM6 or RBM5 mRNA expression levels.

## Results

### Identification of three novel RBM6-RBM5 chimeric transcripts

**Figure 1:**
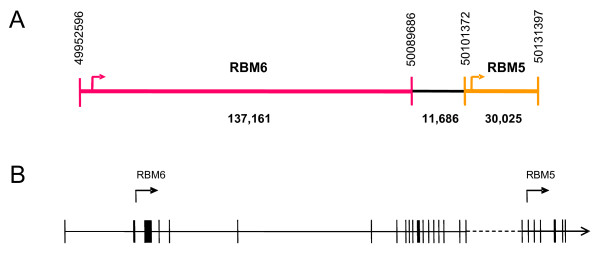
**Genomic organisation of *RBM6 *and *RBM5***. **A**. Diagram outlining the orientation and spatial relationship between *RBM6 *and *RBM5*. The size of both genes, in relation to the distance between them, is indicated in base-pairs. Vertical numbers represent chromosomal markers. Sideways arrows represent translation start sites. **B**. Scale diagram comparing *RBM6 *intragenic distances to the *RBM6/RBM5 *intergenic distance. Vertical bars represent exons, solid horizontal lines represent introns and the dotted line represents the intergenic region. The sideways arrows represent the translation start sites, within exon 2 of both genes.

**Figure 2:**
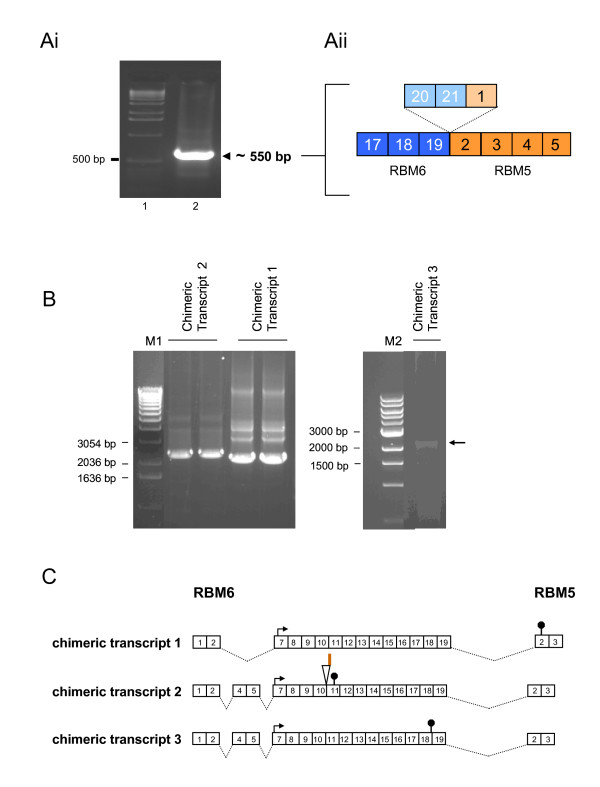
**Identification of an RBM6-RBM5 chimeric transcript by nested RT-PCR. Ai**. Agarose gel of the nested RT-PCR result from MDA-MB-231 mRNA, using outer primers RBM6E8F and RBM5E7R and inner primers RBM6E17F and RBM5E4R, showing the ~550 bp amplicon in lane 2. Lane 1: DNA ladder (1 kb, Invitrogen). **Aii**. Schematic of the amplified 550 bp fragment, showing the splicing out of RBM6 exons 20 and 21, and RBM5 exon 1. Blue boxes indicate exons of RBM6, orange boxes indicate exons of RBM5, dotted lines delineate site of intragenic splicing. **B**. Agarose gel of the nested RT-PCR results for full-length RBM6-RBM5 chimeric transcripts, using outer primers RBM6Fb and RBM5E7R and inner primers RBM6Fc and RBM5E5R. The three chimeric transcripts are from different experiments. Chimeric transcripts 1 and 3 were amplified from Jurkat cells, whereas chimeric transcript 2 was amplified from skeletal muscle tumour. M1: 1 kb DNA ladder (Invitrogen). M2: 1 kb DNA ladder (New England Biolabs). Arrow identifies the faint amplicon observed for chimeric transcript 3. **C**. Schematic detailing the splicing patterns associated with the three chimeric transcripts. Numbered boxes represent exons, dotted lines represent alternative splice sites. Arrows: delineate putative translation start codons of longest ORFs. Stop signs: represent putative translation stop codons of the longest ORFs. The triangle in chimeric transcript 2 indicates the site of the 17 nucleotide insertion (represented by the red line) from RBM6 intron 10. Not drawn to scale.

*RBM6 *and *RBM5 *map to the short arm of human chromosome 3 and are separated by ~11 kb (Figure 1). Since transcription-induced chimeras can be generated from genes which are physically located up to 50 kb from each other (the median distance being ~8.5 kb [2]), we decided to investigate the existence of such a chimeric transcript from this locus. Nested PCR was performed on cDNA generated from total RNA from the human breast adenocarcinoma cell line MDA-MB-231. The outer nested primers were located within exon 8 of RBM6 and exon 7 of RBM5, and the inner nested primers were located within exon 17 of RBM6 and exon 4 of RBM5. In order to eliminate artifacts from template switching events (which are normally attributed to sequence similarities at intron boundaries), thermostable reverse transcriptase was used [21]. An ~550 base-pair (bp) amplicon was observed (Figure 2Ai), which sequencing revealed lacked exons 20 and 21 of RBM6 and exon 1 of RBM5 (Figure 2Aii).

Having determined, by nested RT-PCR, that an RBM6-RBM5 chimeric transcript did exist, we then focused on obtaining a full-length open reading frame. Since during the course of our investigations we found that the amplicon was expressed most highly in the human Jurkat T lymphoblastic leukemia cell line and in human skeletal muscle tumour tissue, we used total RNA from Jurkat cells and skeletal muscle tumour as templates. Outer nested primers specific for exon 1 of RBM6 and exon 7 of RBM5 were used in combination with inner nested primers specific to exon 1 of RBM6 and exon 5 of RBM5. The experiment was repeated several times. Two different amplicons were identified in the Jurkat cells (chimeric transcripts 1 and 3, Figure 2B), while a third unique amplicon was identified in the skeletal muscle tumour (chimeric transcript 2, Figure 2B). All amplicons were sequenced. The different transcripts were termed RBM6-RBM5 chimeric transcript 1 (Jurkat cell origin), RBM6-RBM5 chimeric transcript 2 (skeletal muscle origin) and RBM6-RBM5 chimeric transcript 3 (Jurkat cell origin). The common characteristic of all three chimeric transcripts was a lack of exons 3, 6, 20, and 21 of RBM 6, and exon 1 of RBM5. The difference between all three chimeric transcripts related to the presence or absence of exons 4 and 5 and a 17 nt insertion from RBM6 intron 10: chimeric transcript 1 lacked RBM6 exons 3 to 6; chimeric transcript 2 lacked RBM6 exon 3 and exon 6, and included the additional 17 nt from RBM6 intron 10, and; chimeric transcript 3 only lacked RBM6 exon 3 and exon 6 (Figure 2C).

### Differential expression of the RBM6-RBM5 chimeric transcripts in non-tumour and tumour tissue

To this point, RBM6-RBM5 chimeric expression was observed in the human breast adenocarcinoma cell line MDA-MB-231, T lymphoblastic leukemia Jurkat cell line and skeletal muscle tumour, all representing malignant cancers. We therefore decided to investigate the relationship between RBM6-RBM5 chimeric expression and malignancy by examining expression in non-malignant tissue.

**Figure 3:**
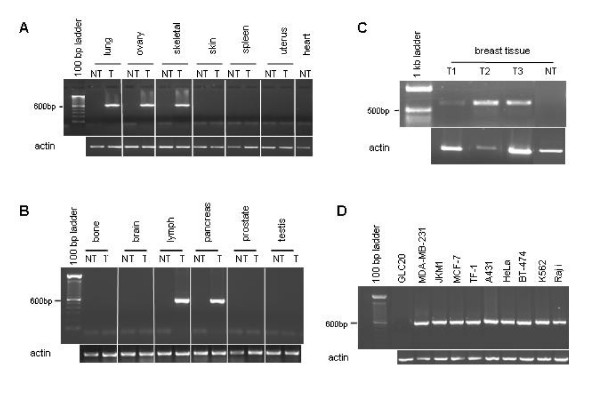
**RBM6-RBM5 chimeric transcript expression**. Chimeric expression was examined using nested RT-PCR on tissue or cell line mRNA. **A**. Expression in purchased human tissue samples: lung – large cell carcinoma; ovary – cystadenoma; skeletal muscle – malignant fibrous histiocytoma, poorly differentiated; skin – malignant melanoma; spleen – malignant lymphoma; uterus – adenocarcinoma, well differentiated. **B**. Expression in purchased human tissue samples: bone – osteoblastic osteosarcoma; brain – astrocytoma, moderately differentiated; lymph node – non-Hodgkin's lymphoma of tonsil; pancreas – insulinoma; prostate – adenocarcinoma, moderately differentiated; and testis – seminoma. **C**. Expression in three tumour samples from the Ontario Cancer Research Network, and an unmatched purchased non-tumour sample. **D**. Expression in human cancer cell lines: GLC20 – small cell lung carcinoma; MDA-MB-231 – pleural effusion of a breast adenocarcinoma; JKM1 – Jurkat T lymphoblastic leukemia subclone; MCF-7 – pleural effusion of a breast adenocarcinoma; TF-1 – erythroblastic leukemia; A431 – epidermoid carcinoma; HeLa – cervical carcinoma; BT-474 – invasive ductal breast carcinoma; K-562 – chronic myelogenous leukemia; Raji – Burkitt's lymphoma. NT: non-tumour tissue, T: tumour tissue.

Nested PCR was carried out in various tissues, including non-tumour and tumour, using the same primers as mentioned above to identify the ~550 bp amplicon. (Note, since the PCR product generated by these primers was common to all three transcripts, this experiment did not discriminate between the specific chimeric transcripts.) Interestingly, chimeric expression was never observed in non-tumour tissue samples. In addition, expression was not consistently observed in all tumour tissue types: expression was observed in lung, ovary, skeletal muscle, lymph node, pancreas and breast but not in skin, spleen, uterus, bone, brain, prostate or testis tumour tissue (Figure 3A, B and 3C). Numerous cancer cell lines were also examined for expression of the chimeric transcripts, including breast (MDA-MB-231, MCF-7, BT-474), hematopoietic (Jurkat subclone JKM1, TF-1, Raji, K562), lung (GLC20), skin (A431) and cervical (HeLa). GLC20 is a small cell lung cancer cell line known to harbour a 440 kb homozygous deletion at 3p21.3 [22], and was therefore selected as a negative control for RBM6-RBM5 chimeric transcript detection. Nested RT-PCR results indicated that RBM6-RBM5 chimeric transcription occurred in all cancer cell lines, irrespective of tissue of origin, excluding the GLC20 negative control.

Further analysis of RBM6-RBM5 transcription-induced chimeric expression in tumour samples revealed that expression in the tumour tissue was not solely dependent on either tumour type or tissue type (Table 1). For instance, two different large cell lung carcinomas had different chimeric profiles, demonstrating not only that not all lung tumours are positive for chimeric expression, but that not all lung tumours of the same type (large cell), are positive. Likewise for the malignant fibrous histiocytoma of skeletal muscle, non-Hodgkin's lymphoma, insulinoma of the pancreas and cystadenoma of the ovary. These results suggest that some additional factor, such as tumour grade or differentiation status, plays a role in RBM6-RBM5 chimeric expression. Unfortunately, insufficient pathological information was available for the lung, ovarian, skeletal muscle, lymph or pancreatic tumours to evaluate possible criteria which might account for expression differences. Fortunately, the breast tumour samples were provided with more pathological information (refer to Table 2). There were no apparent expression differences related to grade, lymph node involvement, estrogen or progesterone receptor status, HER2 status or donor age; however, there appeared to be a threshold tumour size after which RBM6-RBM5 chimeric transcript expression was detectable (Table 2). All three chimeric transcript expressing breast tumour samples (#1, 2, 3) were the largest in our five-sample cohort (4.5, 6.0 and 3.0 cm in the largest dimension, respectively). The sample with inconsistent chimeric transcript expression was 2.7 cm in its largest dimension, and the chimeric transcript negative tumour was 2.5 cm in its largest dimension. This observation suggests that tumour size, as a measure of breast tumour differentiation status, may be an important parameter of RBM6-RBM5 chimeric expression. A larger sample size, however, is required to confirm this hypothesis.

**Table 1:** Summary of expression of RBM6-RBM5 chimeric transcripts in various tumour and non-tumour tissues

	Breast	Lung	Lymph node	Pancreas	Ovary	Skeletal muscle
Non-tumour 1	-	-	-	-	-	-
Non-tumour 2	-	-	-	-	-	-
Non-tumour 3	-	-	-	-	-	-
Tumour 1	+	+ ^a^	+ ^c^	+ ^e^	+ ^f^	+ ^h^
Tumour 2	+	- ^a^	- ^c^	- ^e^	- ^f^	- ^h^
Tumour 3	+	- ^b^	- ^d^		- ^g^	
Tumour 4	+/-					
Tumour 5	-					

a: large cell carcinoma

b: squamous cell carcinoma

c: non-Hodgkin's lymphoma

d: T cell Hodgkin's lymphoma

e: insulinoma

f: cystadenoma

g: adenocarcinoma, poorly differentiated

h: malignant fibrous histiocytoma, pooly differentiated

(+): positive for expression; (-): negative for expression. Blank grids indicate no corresponding cDNA was examined

**Table 2:** Clinicopathological parameters of breast tumour samples

Tumour sample	Chimeric transcript status	Tumour grade	Lymph node metastases	Estrogen receptor status	Progesterone receptor status	HER2 status	Patient age (yrs)	Tumour size (cm)
T1	+	3	+	+	-	+	33	4.5
T2	+	3	+	+	+	?	81	6.0
T3	+	2	+	+	-	-	71	3.0
T4	+/-	3	+	+	+	-	54	2.7
T5	-	2	+	+	+	?	86	2.5

+ : positive expression

- : negative expression

? : indeterminate expression

### Protein analysis of the RBM6-RBM5 chimeric transcripts

While evidence points to the fact that transcription-induced chimerism occurs quite frequently in the human genome [2], only a few fusion proteins have actually been identified, only a portion of which have a known function [23-25].

Sequence analysis of the three chimeric transcripts revealed the longest ORF initiating, for each, within exon 7 of RBM6 but terminating at different premature termination codons (PTC) in each of the three transcripts (Figure 2C). In chimeric transcript 1, the PTC occurred within exon 2 of RBM5, resulting in a putatively ~62 kDa chimeric protein of 521 amino acids (aa), in frame with RBM6 but including an additional four aa from RBM5: the 5'-untranslated region (UTR) was 186 nt long. Chimeric transcripts 2 and 3 both contained PTCs situated in RBM6, putatively encoding two novel, truncated RBM6 proteins. The presence of the intron 10 insertion in chimeric transcript 2 created a PTC located within exon 11 of RBM6, generating an ORF of 199 aa, putatively encoding an ~24 kDa protein with high homology to RBM6 but with six novel, additional aa from the 17 nt insertion. Chimeric transcript 3 contained a point mutation in exon 18, generating a PTC and thus resulting in an ORF putatively encoding an ~58 kDa protein of 482 aa. The 5'-UTR of chimeric transcripts 2 and 3 was 346 nt. Significantly, the presence of long 5'-UTRs (>100 nt) and premature termination codons in each of these long ORFs, and the lack of a Kozak sequence surrounding the exon 7 ATG codon [26] suggested that either translation initiation would be inhibited or the chimeric transcripts would be degraded by nonsense-mediated decay [27,28]. If translation initiated within RBM6 exon 2 for each of the chimeric transcripts, thereby utilizing the partial Kozak sequence-associated translation initiation codon for RBM6 protein, premature termination would occur in all three transcripts. In chimeric transcript 1 the PTC would occur within RBM6 exon 7, resulting in a putatively ~2 kDa protein of 17 aa. In chimeric transcripts 2 and 3 the PTCs would occur within RBM6 exon 4, resulting in a putatively ~4 kDa protein of 33 aa. Since premature termination codons were noted for all of the above described open reading frames, we postulated that no protein product, particularly no RBM6-RBM5 "fusion" protein, would actually be encoded by these novel chimeric transcripts.

**Figure 4:**
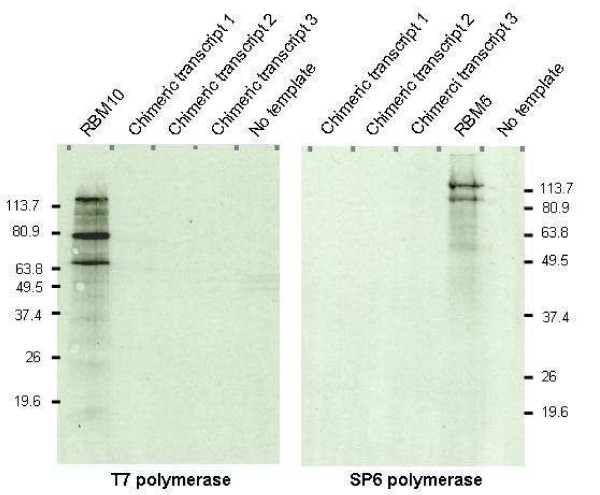
***In vitro *transcription/translation of chimeric transcript cDNAs**. *In vitro *transcription/translation results using the T7 and SP6 TNT ^®^quick coupled transcription/translation system in the presence of [^35^S] methionine. Products were electrophoresed through a denaturing polyacrylamide gel. pcDNA3.RBM10 and the antisense pcDNA3.RBM5(-) constructs were used as positive controls for the T7 and SP6 polymerases, respectively. Reaction minus template ("no template") was used as a negative reaction control.

To investigate this, we examined protein production from each of the chimeric transcripts. Since the mRNA expression levels were relatively low (only being detectable using nested RT-PCR), an *in vitro *transcription/translation system was used rather than an immunoblot technique. Chimeric cDNAs were first cloned into the pCR^®^II TOPO^® ^vector, then incubated in a rabbit reticulocyte lysate with radioactive methionine. As shown in Figure 4, no polypeptide products were observed for any of the chimeric transcripts, from either orientation. Positive controls included pcDNA3.RBM10 for the T7-specific reaction, producing the expected product of ~120 kDa (and two additional products, always visualized), and the antisense pcDNA3.RBM5(-) construct for the SP6-specific reaction, producing the expected ~115 kDa major product. These results suggested that the RBM6-RBM5 chimeric transcripts are non-coding mRNAs.

### RBM6-RBM5 chimeric transcript expression in relation to RBM6 and RBM5

Since no RBM6-RBM5 protein product was observed, it was tempting to speculate that either (1) the novel chimeric transcripts function at the mRNA level, or (2) the chimeric RNA's are "non-functional", but the physical act of chimeric transcription functions to inhibit, or at least downregulate, expression of the two individual genes, *RBM6 *and *RBM5*. We therefore initiated our investigation by examining the relationship between expression of RBM6 and RBM5 in tumour samples that were either chimeric positive or negative. If RBM6-RBM5 non-coding mRNAs are indeed non-functional, and the physical act of chimeric expression is involved in the regulation of *RBM6 *and/or *RBM5 *expression, then expression of both genes in the chimeric positive tumours would be expected to decrease in relation to the chimeric negative tumours. If, however, RBM6-RBM5 non-coding mRNAs are indeed functional, then the expected outcome on *RBM6 *and/or *RBM5 *expression would be less predictable.

**Figure 5:**
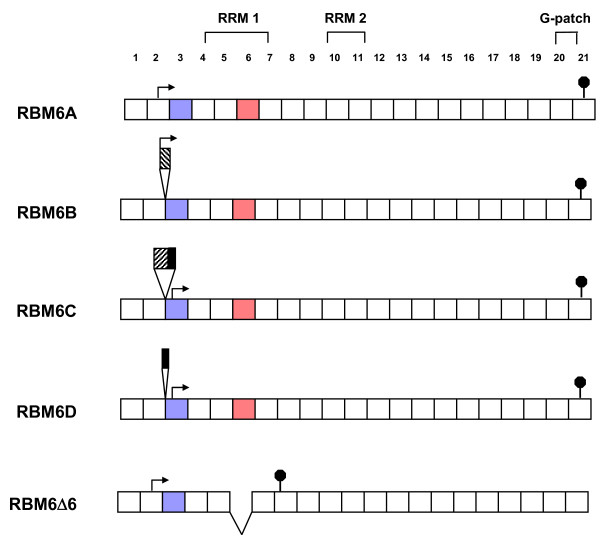
**Alternative splice variants of RBM6**. The five RBM6 alternative splice variants, showing deleted exons, differentially inserted regions from within intron 2, putative translation start sites (arrows) and putative stop codons (stop signs) [7]. Boxes represent exons and are not drawn to scale. Exon 3 (which is present in all RBM6 variants but absent in all RBM6-RBM5 chimeric transcripts) is depicted in blue, while exon 6 (which is differentially spliced in RBM6 variants and the RBM6-RBM5 chimeras, and contains the first of two RRM domains) is depicted in red. The forward and backward hatched boxes drawn as alternatively spliced sequences from intron 2, represent two different sequences, while the solid black box represents the same sequence in both RBM6C and RBM6D.

SYBR green based quantitative PCR (QPCR) technology was used in the analyses. Since lack of exon 3 was uniquely associated with the RBM6-RBM5 transcription-induced chimeras (refer to Figure 2C and Figure 5), exon 3-specific primers were used to differentiate between non-chimeric and chimeric RBM6 transcripts. Likewise, RBM5 exon 1-specific primers were used to differentiate between non-chimeric and chimeric RBM5 transcripts. Two paired skeletal muscle samples were chosen, one that expressed the chimeric transcripts and one that did not. Two breast tumour tissue samples were also chosen, one that expressed the chimeric transcripts (T1, Figure 3C) and one that did not. Commercial normal breast cDNA was used for normalization.

Interestingly, the results showed that elevated levels of chimeric transcript were in fact associated with elevated levels of RBM6 and RBM5. RBM6 was highly expressed in the chimeric positive skeletal muscle tumour compared to the chimeric negative skeletal muscle tumour. The raw data are presented as Table 3, showing an ~1.5-fold increase of RBM6 mRNA expression levels in the chimeric negative tumour sample (compared to non-tumour sample), but an ~14-fold increase of RBM6 in the chimeric positive tumour sample (compared to non-tumour sample). A similar trend was observed in the breast tissues (an ~16-fold increase in RBM6 expression in the chimeric negative tumour sample and an ~25-fold increase in the chimeric positive tumour sample), supporting an expression correlation between RBM6 expression and that of the RBM6-RBM5 chimeras, and demonstrating that the phenomenon was not tissue-specific.

**Table 3:** Relative expression levels of RBM6 in chimeric positive versus chimeric negative tumour samples compared to non-tumour

	RBM6 (mean ± SD)	Control (S28) (mean ± SD)	normalized RBM6	fold change in tumour compared to non-tumour
**Breast Tissue**				
				
tumour				
chimeric transcript (+)	0.403 ± 0.008	0.073 ± 0.007	5.521	24.43
chimeric transcript (-)	0.243 ± 0.012	0.066 ± 0.01	3.682	16.3
non-tumour	0.051 ± 0.006	0.226 ± 0.06	0.226	1
				
**Skeletal Muscle**				
				
tumour: chimeric transcript (+)	0.0488 ± 0.001	0.0489 ± 0.005	0.998	14.06
non-tumour	0.005 ± 0.0003	0.070 ± 0.01	0.071	1
tumour: chimeric transcript (-)	0.063 ± 0.01	0.297 ± 0.058	0.212	1.52
non-tumour	0.022 ± 0.002	0.158 ± 0.02	0.139	1

(+) : chimeric transcript positive, (-) : chimeric transcript negative.

One interpretation of the observation of a positive correlation between RBM6-RBM5 chimeric expression and RBM6 expression is that chimeric expression represents a small fraction of tumour-associated aberrant "run-off" or "leakage" *RBM6 *transcription events [2]. If this were the case, one might still expect to see a decrease in RBM5 mRNA expression, since the promoter of RBM5 is inoperable due to chimera-associated splicing. As shown in Table 4, however, RBM5 mRNA expression levels, in breast tissue, were found to be higher in chimeric positive tumours than chimeric negative tumours, further suggesting that the process of transcription-induced chimerism at this locus is not responsible for repressing expression of either of the two putative tumour suppressor genes *RBM6 *and *RBM5*. It is therefore hypothesized that the three novel RBM6-RBM5 transcription-induced chimeras are functional non-coding RNAs, whose role is not one of *RBM6 *and/or *RBM5 *transcription regulation.

**Table 4:** Relative expression levels of RBM5 in chimeric positive versus chimeric negative tumour samples compared to non- tumour

	RBM5 (mean ± SD)	Control (S28) (mean ± SD)	normalized RBM5	fold change in tumour compared to non-tumour
**Breast Tissue**				
				
tumour				
chimeric transcript (+)	0.073 ± 0.001	0.013 ± 0.001	5.615	35.31
chimeric transcript (-)	0.041 ± 0.005	0.029 ± 0.004	1.413	8.886
non-tumour	0.017 ± 0.003	0.107 ± 0.005	0.159	1

(+) : chimeric transcript positive, (-) : chimeric transcript negative.

## Discussion

We previously reported that RBM6 mRNA expression was significantly upregulated in human breast tumour tissue compared to non-tumour tissue [6]. Here we report that RBM6 mRNA expression was also elevated in skeletal muscle tumour tissue compared to normal, from ~1.5-fold in one tumour to ~14-fold in a different tumour. The larger increase in RBM6 expression levels was associated with the expression of three novel RBM6-RBM5 transcription-induced chimeras, each lacking exons 3, 6, 20, and 21 of RBM6 and exon 1 of RBM5, but differing in the presence or absence of exon 4, exon 5 and a 17 nt sequence from intron 10 of RBM6. All three transcripts incorporated the canonical splice sites from both genes (excluding the 17 nt intron 10 insertion). According to Akiva and colleagues [2], the most abundant transcription-induced chimeric splicing pattern, occurring in 80 % of the events, removes any exon of the upstream gene and the first exon of the downstream gene. Each of the three novel RBM6-RBM5 transcription-induced chimeras falls within this category, and is, therefore, not a rare form of "chimerism".

The RBM6-RBM5 chimeric transcripts appear to be differentially expressed in tumour compared to non-tumour samples. While no expression was detected in non-tumour samples, chimeric transcripts were observed in carcinoma (breast, lymph node, lung, ovary and pancreas) and sarcoma (skeletal muscle) samples. The differential chimeric expression patterns observed in tumours of the same tissue type, for instance non-Hodgkin's versus T cell Hodgkin's lymphoma or large cell versus squamous cell lung carcinoma, may not reflect tumour cell origin-specific expression patterns so much as the differentiation status of that individual tumour sample. This hypothesis is supported by our observations in the breast tumour samples, where chimeric expression appeared to be associated with a threshold tumour size.

For the three novel RBM6-RBM5 transcription-induced chimeric transcripts identified, it was interesting to note that each was generated by differential splicing of exons incorporating putative functional consensus sequences, e.g., the novel 20-repeat hexamer sequence within exon 3 (hypothesized to play a role in RNA interactions [7]), the RNA recognition motif (RRM) within exon 6 (an RNA binding domain [29]), and the G-patch domain associated with exon 20 (involved in RNA splicing [30]). In addition, differential splicing within RBM6 intron 10 in chimeric transcript 2 resulted in elimination of the second RBM6 RRM domain within exon 11. It was therefore interesting to speculate that the transcription-induced chimerism at the RBM6 locus was important to the generation of novel functional RBM6-related proteins with different mechanisms of action; however, no novel fusion protein was generated and there was no reduction in either RBM6 or RBM5 mRNA expression levels associated with RBM6-RBM5 chimeric expression. The consistent splicing patterns associated with the chimeras, all revolving around exons containing putatively significant functional domains, suggests that chimerism at this site, or at least altered expression of RBM6 and perhaps RBM5, is an important and regulated event. The importance of RBM6 tumour-associated expression regulation remains to be determined.

## Conclusion

Transcription-induced chimeras of the neighboring genes *RBM6 *and *RBM5 *were identified in human tumour tissues. No novel fusion proteins were encoded by any of the RBM6-RBM5 chimeras, but chimeric expression was positively correlated with expression of RBM6 and RBM5 mRNA. The functional significance and regulation of this event remain to be elucidated; however, RBM6-RBM5 chimeric transcripts could prove to be useful tumour differentiation markers, although more extensive expression analyses are required to confirm these observations.

GenBank Accession Numbers deposited:

RBM6-RBM5 chimeric transcript 1: EF566883

RBM6-RBM5 chimeric transcript 2: EF566884

RBM6-RBM5 chimeric transcript 3: EF566885

## Methods

### Cell lines and tissue samples

RNA from the following cell lines was used to generate the cDNA for PCR expression studies: GLC20 (generously provided by Charles Buys, Gröningen University, The Netherlands), MDA-MB-231 (ATCC# HTB-26), Jurkat (JKM1) [31], MCF-7 (the kind gift of David Seldon, Boston University, U.S.A.), TF-1 (ATCC# CRL-2003), HeLa (provided by Hoyun Lee, HRSRH) and BT-474 (ATCC # HTB-20). cDNA for the following cell lines was purchased (BioChain Institute, Inc., CA, U.S.A.): A431, K562 and Raji. cDNA for all of the tissue samples, except the breast tumours, was also purchased (BioChain Institute, Inc., CA, U.S.A.). Five breast tumour samples were obtained from the Ontario Cancer Research Network Pilot Distribution Project. Each of these was classified as invasive mammary carcinoma of no special type. Three non-tumour breast samples were purchased (BioChain Institute, Inc., CA, U.S.A.).

### RNA isolation, DNase treatment and reverse-transcription

Total RNA was isolated from the GLC20, MDA-MB-231, JKM1, MCF-7, TF-1, HeLa and BT-474 cell lines, and the breast tumour tissues. RNA was isolated from the cell lines using the RNeasy kit (Qiagen, U.S.A.) and from the breast tissue using TRI-Reagent (Molecular Research Center, Inc., U.S.A.), according to the manufacturer's instructions. For the breast tumour tissue RNA isolation, the tissue and the tissue pulverizer (Beckman) were cooled in liquid nitrogen for 5 min, then 500 mg of tissue were pulverized and dissolved in 1 ml of TRI-Reagent by passing through a series of increasingly smaller-bore needles. Phase separation was achieved with the addition of chloroform, followed by centrifugation. RNA was precipitated from the aqueous layer using isopropanol. RNA pellets were washed with 75% ethanol, air-dried and resuspended in DEPC (Sigma)-treated water. RNA quantity and quality were determined using a bioanalyzer (Agilent Technologies).

To hydrolyze contaminating DNA in the RNA preparations, 1 μg of RNA was incubated with 1 μl of amplification-grade DNase I (Invitrogen) and 1 μl of 10× DNase buffer in a final volume of 10 μl at room temperature for 15 min, then 1 μl of 25 mM EDTA solution was added and the reaction stopped by heating at 65°C for 10 min. Following DNase treatment, 1μg of total RNA was reverse transcribed using the Superscript II kit (Invitrogen), according to the manufacturer's instructions. Briefly, 1 μl of T20-VN (500 ng/μl) and 1 μl dNTP (10 mM) were added to 10 μl of the above DNase treated RNA, incubated at 65°C for 5 min, then chilled on ice. Then, 4 μl 5× first-strand buffer, 2 μl dithiothreitol (DTT), 1 μl RNase Out and 1 μl Superscript reverse transcriptase were added to the reaction. Following a 1 hour incubation, the reaction was stopped by heating at 70°C for 15 min. The newly transcribed cDNA was used directly for PCR amplification. For amplification of the full-length chimeric transcripts, reverse transcription was carried out using the thermostable enzyme supplied with the transcriptor first-strand cDNA synthesis kit (Roche), according to the manufacturer's instructions.

### Primers

The following primers were used, based on GeneBank Accession Numbers NM_005777 (RBM6) and NM_005778 (RBM5):

RBM6Fb: AGGCTGAGGAGAAGGAGGAG

RBM6Fc: AGGAGAAGGAGGAGCGGG

RBM6E8F: GCACCGATCTTCCTGTTCAT

RBM6E17F: GCTGTCAGACCTGCACAAG

QRBM6E3F1: TCTTGGGCGGCAAGACA

QRBM6E3R1: GGCTTCCTGGCAGCCTATG

RBM5E4R: CAGTGACGAGAGGGAGAGCAAGA

RBM5E5R: CGGATGTGAGGCTGATGAAGAGGA

RBM5E7R: TCAAGGAAAGCACATTGCAA

QRBM5E1F1: CGGAGGCGCCATTTTGA

QRBM5E1R1: GAAGCAGCAGTAGCGGTTCTG

### Nested PCR amplification

Two different sets of nested PCR reactions were carried out, one for the identification of a short, internal chimeric product, and the other for the identification and isolation of a chimeric product containing an entire putative ORF. The nested PCR reactions were carried out in an iCycler thermal cycler (BioRad). 2 μl of each cDNA were used as template in a total volume of 50 μl. Reactions contained 200 μm each of deoxynucleoside triphosphate (dATP, dCTP, dGTP and dTTP), 2.5 units of Taq polymerase and 0.2 μM of each primer. For identifying the shorter chimeric transcript, RBM6E8F and RBM5E7R were used as forward and reverse primers, respectively, in the first round of amplification. First round amplification was carried out at 95°C for 3 min, followed by 35 cycles of 94°C for 30 sec, 55°C for 30 sec and 72°C for 2 min 20 sec, followed by 72°C for 10 min. The second round of amplification was carried out using RBM6E17F and RBM5E4R as forward and reverse primers, respectively, with 2 μl of the first round PCR reaction as template. This second round of amplification was performed at 95°C for 3 min, followed by 40 cycles of 94°C for 30 sec, 55°C for 30 sec and 72°C for 45 sec, followed by 72°C for 10 min. Electrophoresis of the PCR products was performed through a 2% agarose gel containing 0.1 μg/ml ethidium bromide.

For the longer chimeric transcripts, RBM6Fb and RBM5E7R were used as forward and reverse primers, respectively, for the first round of amplification. The first round of amplification was carried out at 95°C for 3 min, followed by 35 cycles of 94°C for 30 sec, 55°C for 30 sec and 72°C for 4 min, followed by 72°C for 10 min. The second round of amplification was carried out by using inner primers RBM6Fc and RBM5E5R, with 2 μl of the first round PCR reaction as template. This second round of amplification was performed at 95°C for 3 min, followed by 40 cycles of 94°C for 30 sec, 55°C for 30 sec and 72°C for 4 min, followed by 72°C for 10 min. Electrophoresis of the PCR products was performed through a 0.8 % agarose gel containing 0.1 μg/ml ethidium bromide.

### Quantitative real-time PCR

To determine the relative-fold expression of RBM6 and RBM5 in tumour tissue with or without RBM6-RBM5 chimeric transcript expression, quantitative real-time PCR (QPCR) was performed. RBM6 levels were measured using primers (QRBM6E3F1 and QRBM6E3R1) located within exon 3, since exon 3 sequence was present in each of the known RBM6 RNA splice variants but absent from each of the RBM6-RBM5 chimeric transcripts. RBM5 levels were measured using primers (QRBM5E1F1 and QRBM5E1R1) located within exon 1, since exon 1 sequence was present in each of the known RBM5 RNA splice variants but absent from each of the RBM6-RBM5 chimeric transcripts. Real-time PCR was carried out using SYBR green (Applied Biosystems) technology and an ABI Prism 7900HT Sequence Detection System (Applied Biosystems). In a 25 μl reaction, 12.5 μl of a 2× SYBR Green Master Mix, 7.5 μl of a 2 μM stock of each primer and 5 μl of 1:8 diluted cDNAs were combined. The PCR programme incorporated denaturation at 95°C for 10 min, followed by 40 cycles of amplification at 95°C for 15 sec, 55°C for 15 secs and 72°C for 30 secs. All samples were analyzed in triplicate, and the data were normalized to the S28 internal control.

### TA cloning and sequencing

The three different full-length RBM6-RBM5 chimeric transcripts were amplified by nested PCR, as described above, using the two sets of primers RBM6Fb/RBM5E7R and RBM6Fc/RBM5E5R. The three PCR products, ranging between 2.1–2.4 kb, were gel purified using a gel purification kit (Qiagen) and cloned into the pCR^®^II-TOPO vector using the TOPO TA dual promoter cloning kit (Invitrogen), according to the manufacturer's instructions. The clones obtained were confirmed by sequencing (Mobixlab, McMaster University, Canada), and the sequences compared to GenBank sequences through BLAST [32].

### *In vitro *transcription/translation

The *in vitro *transcription/translation experiments were performed using the T7 and SP6 TNT^® ^Quick Coupled Transcription/Translation Systems (Promega) in the presence of [^35^S] methionine (Perkin Elmer), according to the manufacturer's instructions. Template plasmids were the three pTA constructs, containing the three different chimeric transcript cDNAs, and putatively encoding proteins as large as 62 kDa (chimeric transcript 1), 24 kDa (chimeric transcript 2) and 58 kDa (chimeric transcript 3). The pcDNA3.RBM10 and pcDNA3.RBM5(-) constructs were used as positive controls for the T7 and SP6 polymerases, respectively. 0.5 μg of each plasmid was used per 25 μl reaction, which was incubated at 30°C for 90 min. 7.5 μl of each reaction was separated by 10% SDS-PAGE. Gels were then transferred to PVDF membrane (Pall, Gelman Sciences) and exposed to Hyperfilm (GE Heathcare).

## Competing interests

The author(s) declares that there are no competing interests.

## Authors' contributions

KW participated in the design of the study, performed all the experiments and drafted the manuscript. GU conceived the study and performed the preliminary RNA extraction, cDNA synthesis and nested RT-PCR experiments. LCS oversaw the design of the study, was involved in data interpretation and critically revised the manuscript. All authors have read and approved the final version of the manuscript.
